# Supplementary material for: A Meta‐Analysis of the Effects of Acute Sleep Deprivation on the Cortical Transcriptome in Rodent Models
Source: J Sleep Res. 2025 Oct 1;35(3):e70205. doi: 10.1111/jsr.70205 (PMC13131251; doi:10.1111/jsr.70205)
Supplement: Supplementary file 1 — Data S1: jsr70205‐sup‐0001‐Supinfo.pdf. [file JSR-35-e70205-s003.pdf]

<u>Supplemental Material for:</u>

A Meta-Analysis of the Effects of Acute Sleep Deprivation on the Cortical Transcriptome in Rodent Models

*Cosette A. Rhoads[1], *Megan H. Hagenauer, Ph.D.[2], Jinglin Xiong[1], Erin Hernandez[1], Duy Manh Nguyen[1], Annaka Saffron[3], Elizabeth Flandreau, Ph.D.[4], Stanley Watson Jr., M.D., PhD.[2], Huda Akil Ph.D.[2]

1. Grinnell College, Grinnell, IA USA
2. Michigan Neuroscience Institute, University of Michigan, Ann Arbor, MI USA
3. Johns Hopkins University, Baltimore, MD USA
4. Grand Valley State University, Allendale, MI USA

*Shared first authorship

Direct correspondence to:

Megan Hagenauer, Ph.D.
Michigan Neuroscience Institute
BSRB
109 Zina Pitcher Pl
University of Michigan
Ann Arbor, MI 48109 USA
hagenaue@umich.edu

Supplementary Methods

Data Processing Procedures within Gemma:

Data Preprocessing: Data preprocessing by *Gemma* includes up-to-date microarray probe alignment and RNA-Seq read mapping (Lim et al., 2021a). For RNA-Seq FASTQ files, read adapters are trimmed with Cutadapt (Martin, 2011) and reads aligned with STAR (Dobin et al., 2013) and quantified using RSEM (Li and Dewey, 2011). For microarray data, different probe-to-gene mapping strategies are applied depending on platform. For Affymetrix datasets such as those referenced in our paper, data is extracted from the raw .CEL files using the RMA algorithm (Irizarry et al., 2003) within Affymetrix Analysis Power Tools. Probe sets are collapsed to a single representative sequence, resolving overlaps (Barnes et al., 2005), and aligned to the reference genome with BLAT (Kent, 2002). Alignments are then filtered for specificity, and mapped to transcripts using UCSC Golden Path (Lim et al., 2021a).

Quality Control: For gene-level quality control, the *Gemma* pipeline screens out genes lacking variance in expression levels (<70% distinct values), but otherwise does not apply filtering based on expression level. This inclusion of data from low level expressed genes can increase the risk for false positive and false negative findings in differential expression analyses due to floor effects in transcriptional profiling data, but filtering out low expression data poses its own risks in biasing inferences – in particular, the expression for many neurotransmission-related genes is very low in brain tissue, despite being of particular import for understanding neuropsychiatric conditions (Medina, Hagenauer, Krolewski et al. 2023).

Sample-level quality control is conducted in *Gemma* using a procedure that can be applied similarly across platforms. Samples are considered potential outliers if their adjusted median sample-sample correlation is outside the interquartile range of the sample-sample correlations for the sample with the closest median correlation to them (Lim et al., 2021b). Processing batches are defined automatically using information in the raw data files (*microarrays:* clusters of date stamps, *RNA-Seq:* FASTQ header information such as "device", "run", "flow cell" and/or "lane"), or obtained manually from associated supplementary files or publications, and screened for correlation with the top three principal components of variation in the expression data (Lim et al., 2021b). Batch correction is conducted using an in-house implementation of the ComBat algorithm (Johnson et al., 2007). Batch correction is not performed when no substantial batch effect is detected or when batches are confounded with experimental design factors, in which case the dataset is flagged or split.

Supplemental Tables

Table S1. The full meta-analysis results (16,290 genes, 16,255 stable meta-analysis estimates). *This .xlsx file includes two worksheets: 1) The worksheet "SleepDeprivation_MetaAnalysisResults" provides the full meta-analysis results, with each row representing the results for one gene, and each column providing either gene annotation or meta-analysis statistical output. The results are ordered by p-value, so that the top rows in the worksheet are the genes with the smallest p-values. 2) The worksheet "ColumnDefinitions"*

provides the definitions for the variables present in each column in "SleepDeprivation_MetaAnalysisResults".

Table S2. Full differential expression results from the re-analysis of GSE114845 (19,798 genes). *This .xlsx file includes two worksheets: 1) The worksheet "SleepDep_GSE114845_DEResults" provides the full differential expression results from our re-analysis of GSE114845, with each row representing the results for one gene, and each column providing either gene annotation or differential expression statistical output. The results are ordered by p-value, so that the top rows in the worksheet are the genes with the smallest p-values. 2) The worksheet "ColumnDefinitions" provides the definitions for the variables present in each column in "SleepDep_GSE114845_DEResults".*

	Meta-Analysis: SD									Validation: GSE114845 SD						Exploratory
**Gene Symbol**	**#**	**Log2FC**	**CI_lb**	**CI_ub**	**SE**	**Tstat**	**Pval**	**FDR**		**Expr**	**Log2FC**	**SE**	**Tstat**	**Pval**	**FDR**	**RS Log2FC**
Rimoc1	16	**-0.081**	-0.104	-0.059	0.011	-7.38	1.33E-12	1.96E-08		NA	NA	NA	NA	NA	NA	0.017
Tmod3	18	**-0.111**	-0.151	-0.070	0.021	-5.37	8.00E-08	1.00E-04		4.97	**0.096**	0.021	4.57	1.60E-05	4.18E-05	0.080
Lrrc75a	18	**-0.116**	-0.159	-0.073	0.022	-5.28	1.28E-07	1.15E-04		3.90	**0.077**	0.030	2.60	1.08E-02	1.88E-02	0.078
Mxi1	18	**-0.061**	-0.085	-0.037	0.012	-5.00	5.81E-07	3.79E-04		6.20	**0.040**	0.016	2.50	1.42E-02	2.41E-02	-0.012
Khnyn	18	**-0.091**	-0.130	-0.052	0.020	-4.55	5.38E-06	2.31E-03		4.96	0.009	0.039	0.22	8.23E-01	8.65E-01	0.061
C130071C03Rik	18	**-0.126**	-0.181	-0.070	0.028	-4.44	9.20E-06	3.48E-03		6.20	***0.052***	0.025	2.08	4.02E-02	6.26E-02	***0.114***
Pdk4	18	**-0.171**	-0.248	-0.095	0.039	-4.39	1.12E-05	3.89E-03		3.47	**0.178**	0.047	3.80	2.69E-04	5.95E-04	-0.003
Nrg2	18	**-0.084**	-0.123	-0.046	0.020	-4.30	1.69E-05	5.20E-03		2.72	0.067	0.032	2.10	3.83E-02	6.01E-02	0.072
Rufy3	18	**-0.049**	-0.072	-0.026	0.012	-4.18	2.86E-05	8.17E-03		7.75	0.018	0.010	1.88	6.28E-02	9.42E-02	0.010
Pcdhb3	18	**-0.109**	-0.161	-0.058	0.026	-4.18	2.91E-05	8.17E-03		3.06	0.026	0.063	0.42	6.75E-01	7.41E-01	-0.017
Pqlc3	18	**-0.118**	-0.175	-0.061	0.029	-4.04	5.26E-05	1.09E-02		2.33	**0.227**	0.079	2.87	5.12E-03	9.37E-03	0.097
Rassf4	18	**-0.099**	-0.147	-0.051	0.025	-4.03	5.68E-05	1.13E-02		3.43	0.060	0.045	1.35	1.79E-01	2.41E-01	0.034
Eml1	18	**-0.062**	-0.093	-0.031	0.016	-3.96	7.47E-05	1.36E-02		5.97	0.009	0.015	0.59	5.55E-01	6.31E-01	0.050
Fam13c	18	**-0.081**	-0.121	-0.040	0.020	-3.94	8.30E-05	1.47E-02		5.98	**0.110**	0.022	5.09	2.04E-06	5.88E-06	0.051
Slain1	16	**-0.083**	-0.125	-0.042	0.021	-3.93	8.48E-05	1.47E-02		5.17	-0.039	0.022	-1.79	7.70E-02	1.13E-01	-0.022
Sc5d	18	**-0.090**	-0.135	-0.045	0.023	-3.91	9.05E-05	1.52E-02		5.75	0.054	0.055	0.98	3.30E-01	4.08E-01	0.052
Acadm	18	**-0.081**	-0.122	-0.040	0.021	-3.90	9.50E-05	1.56E-02		4.71	-0.006	0.014	-0.42	6.77E-01	7.42E-01	0.042
Rufy2	18	**-0.055**	-0.083	-0.027	0.014	-3.85	1.17E-04	1.75E-02		6.79	-0.035	0.018	-1.91	5.92E-02	8.93E-02	0.018
Septin8	18	**-0.056**	-0.084	-0.027	0.014	-3.85	1.20E-04	1.77E-02		8.56	**0.074**	0.014	5.11	1.89E-06	5.49E-06	0.049
Ccdc30	18	**-0.067**	-0.101	-0.032	0.018	-3.78	1.57E-04	2.09E-02		4.13	**0.044**	0.018	2.46	1.58E-02	2.66E-02	0.047
Mogs	18	**-0.081**	-0.124	-0.039	0.022	-3.76	1.69E-04	2.20E-02		4.43	**0.055**	0.019	2.86	5.28E-03	9.65E-03	0.069
Car2	18	**-0.112**	-0.171	-0.054	0.030	-3.75	1.74E-04	2.23E-02		7.24	-0.091	0.048	-1.89	6.22E-02	9.33E-02	-0.032
Bean1	18	**-0.110**	-0.168	-0.052	0.029	-3.74	1.83E-04	2.29E-02		3.95	-0.001	0.026	-0.04	9.72E-01	9.80E-01	0.039
Tal1	18	**-0.141**	-0.214	-0.067	0.038	-3.74	1.86E-04	2.29E-02		0.86	0.068	0.051	1.33	1.88E-01	2.51E-01	0.144
Rabl2	18	**-0.074**	-0.113	-0.035	0.020	-3.72	1.96E-04	2.38E-02		4.79	0.032	0.025	1.32	1.89E-01	2.53E-01	0.086
Cep120	18	**-0.042**	-0.065	-0.020	0.011	-3.69	2.20E-04	2.65E-02		5.70	0.017	0.013	1.24	2.17E-01	2.85E-01	-0.030
Nol4l	18	**-0.085**	-0.131	-0.039	0.023	-3.65	2.61E-04	2.97E-02		5.85	**0.092**	0.023	4.05	1.10E-04	2.57E-04	0.083
Pnrc1	18	**-0.155**	-0.238	-0.071	0.042	-3.64	2.72E-04	3.03E-02		4.47	0.005	0.024	0.19	8.50E-01	8.86E-01	0.068
BC002059	18	**-0.158**	-0.245	-0.072	0.044	-3.59	3.35E-04	3.59E-02		2.70	**0.067**	0.025	2.71	8.08E-03	1.43E-02	-0.023
Zfp658	18	**-0.185**	-0.286	-0.083	0.052	-3.57	3.55E-04	3.73E-02		3.11	**0.341**	0.106	3.23	1.76E-03	3.46E-03	0.169
Srpk1	18	**-0.049**	-0.077	-0.022	0.014	-3.54	3.94E-04	4.01E-02		6.56	0.000	0.015	-0.03	9.77E-01	9.83E-01	0.061
Efnb2	18	**-0.049**	-0.076	-0.022	0.014	-3.52	4.30E-04	4.20E-02		5.75	***-0.038***	0.018	-2.10	3.84E-02	6.02E-02	-0.013
2410021H03Rik	16	**-0.108**	-0.168	-0.048	0.031	-3.52	4.38E-04	4.25E-02		-0.15	0.090	0.092	0.97	3.35E-01	4.14E-01	0.194
Sncaip	18	**-0.100**	-0.156	-0.043	0.029	-3.48	5.10E-04	4.75E-02		1.72	-0.049	0.043	-1.12	2.67E-01	3.41E-01	0.100
Mtmr14	18	**-0.065**	-0.102	-0.028	0.019	-3.46	5.32E-04	4.90E-02		3.69	-0.030	0.025	-1.18	2.43E-01	3.14E-01	0.036
Zfp354a	18	**-0.089**	-0.140	-0.039	0.026	-3.46	5.50E-04	4.95E-02		3.00	-0.030	0.028	-1.07	2.89E-01	3.65E-01	-0.026
Dtx4	18	**-0.081**	-0.126	-0.035	0.023	-3.45	5.58E-04	4.99E-02		5.98	**0.070**	0.017	4.08	9.87E-05	2.32E-04	0.075

Table S3. Genes that were downregulated in the murine cortex following SD in our meta-analysis of public transcriptional profiling data (FDR<0.05, collective n=293) but not validated by a separate large RNA-Seq study (GSE114845, n=86 samples representing 222 mice). *Differential expression was considered to be not validated if 1) the opposite direction of effect was observed in GSE114845 than what was observed in the meta-analysis or 2) the same direction of effect was observed in GSE114845 as in the meta-analysis, but the differential expression was not significant (FDR<0.05). Similar to **Table 2**, within the Log(2)Fold Change (Log2FC) columns, blue is used to highlight down-regulation, pink is used to highlight up-*

regulation, bold text indicates FDR<0.05, and bold/italic text indicates nominal significance (p<0.05). *Column definitions: #= Number of differential expression results (SD vs. control statistical contrasts) that contributed to the meta-analysis estimate for that gene; Log2FC=estimated SD vs. control Log2 Fold Change; CI_lb & CI_ub=Lower and upper bound for the 95% confidence interval for the Log2FC; SE=Standard error for the Log2FC, Tstat=T-statistic for the Log2FC, Pval=Nominal p-value for the Log2FC, FDR=False discovery rate for the Log2FC (q-value or adjusted p-value), Expr=Average Log2 expression (counts per million: cpm) for the gene, RS Log2FC: estimated moderating effect (Log(2) Fold Change) of recovery sleep (RS) on the effect of SD within our exploratory meta-analysis.*

	Meta-Analysis: SD								Validation: GSE114845 SD						Exploratory
Gene Symbol	#	Log2FC	CI_lb	CI_ub	SE	Tstat	Pval	FDR	Expr	Log2FC	SE	Tstat	Pval	FDR	RS Log2FC
Thg1l	18	**0.103**	0.074	0.132	0.015	7.01	2.41E-12	1.96E-08	3.90	0.052	0.041	1.27	2.07E-01	2.73E-01	-0.064
Fancf	18	**0.109**	0.071	0.147	0.019	5.61	2.07E-08	6.75E-05	0.95	**-0.249**	0.050	-4.95	3.57E-06	1.00E-05	-0.043
Gm14285	18	**0.182**	0.116	0.248	0.034	5.39	6.95E-08	1.00E-04	-0.78	0.088	0.136	0.64	5.21E-01	6.00E-01	-0.062
Exosc4	18	**0.146**	0.093	0.200	0.027	5.37	7.73E-08	1.00E-04	3.57	**-0.062**	0.025	-2.51	1.38E-02	2.35E-02	-0.026
4931429L15Rik	18	**0.136**	0.086	0.186	0.026	5.31	1.09E-07	1.11E-04	NA	NA	NA	NA	NA	NA	-0.014
Card6	18	**0.145**	0.080	0.210	0.033	4.36	1.31E-05	4.37E-03	1.13	-0.042	0.040	-1.05	2.95E-01	3.71E-01	***-0.160***
Tmem81	18	**0.132**	0.072	0.191	0.030	4.33	1.51E-05	4.84E-03	1.59	**-0.142**	0.051	-2.77	6.90E-03	1.24E-02	-0.063
Cspg4	18	**0.117**	0.062	0.173	0.028	4.16	3.17E-05	8.62E-03	4.55	0.022	0.031	0.72	4.72E-01	5.52E-01	-0.112
Jund	18	**0.083**	0.044	0.122	0.020	4.15	3.34E-05	8.78E-03	6.44	0.043	0.037	1.18	2.43E-01	3.14E-01	-0.047
Bphl	18	**0.060**	0.032	0.089	0.015	4.13	3.56E-05	9.07E-03	3.88	**-0.077**	0.024	-3.24	1.68E-03	3.31E-03	0.000
Lsm3	18	**0.110**	0.057	0.162	0.027	4.10	4.16E-05	9.98E-03	3.21	0.048	0.027	1.80	7.46E-02	1.10E-01	-0.103
Gemin6	18	**0.122**	0.064	0.181	0.030	4.09	4.33E-05	1.00E-02	1.66	-0.040	0.033	-1.22	2.25E-01	2.93E-01	-0.085
Sh3bp2	18	**0.110**	0.057	0.163	0.027	4.05	5.21E-05	1.09E-02	1.44	0.065	0.049	1.34	1.83E-01	2.45E-01	-0.011
Psmc2	18	**0.052**	0.026	0.077	0.013	3.99	6.59E-05	1.23E-02	6.23	-0.016	0.019	-0.86	3.90E-01	4.71E-01	-0.034
Maip1	18	**0.084**	0.042	0.125	0.021	3.95	7.95E-05	1.42E-02	4.16	0.014	0.018	0.79	4.32E-01	5.13E-01	-0.014
Znhit3	18	**0.081**	0.041	0.122	0.021	3.91	9.05E-05	1.52E-02	3.71	0.025	0.022	1.14	2.59E-01	3.32E-01	0.008
Rhob	18	**0.107**	0.052	0.162	0.028	3.84	1.21E-04	1.77E-02	7.28	0.025	0.024	1.05	2.97E-01	3.73E-01	***-0.140***
Clec18a	18	**0.129**	0.063	0.195	0.034	3.81	1.39E-04	1.89E-02	3.29	0.064	0.064	1.00	3.22E-01	4.00E-01	0.012
Gstm6	18	**0.126**	0.060	0.191	0.034	3.75	1.76E-04	2.24E-02	1.04	0.103	0.060	1.71	9.14E-02	1.32E-01	-0.030
Nsmce3	18	**0.075**	0.036	0.114	0.020	3.74	1.82E-04	2.29E-02	4.19	-0.028	0.022	-1.24	2.19E-01	2.86E-01	***-0.096***
Gm6225	16	**0.276**	0.131	0.420	0.074	3.74	1.87E-04	2.29E-02	1.92	-0.016	0.038	-0.41	6.85E-01	7.48E-01	-0.241
Tprn	18	**0.095**	0.043	0.146	0.026	3.61	3.11E-04	3.42E-02	3.58	0.007	0.030	0.24	8.10E-01	8.55E-01	-0.115
Hrob	18	**0.103**	0.047	0.158	0.029	3.59	3.26E-04	3.54E-02	0.28	0.025	0.071	0.35	7.29E-01	7.86E-01	-0.082
Kcng2	18	**0.172**	0.077	0.267	0.049	3.55	3.82E-04	3.96E-02	1.47	**-0.224**	0.058	-3.87	2.06E-04	4.62E-04	-0.073
Gjc1	18	**0.141**	0.063	0.220	0.040	3.54	4.07E-04	4.06E-02	2.21	0.062	0.036	1.73	8.72E-02	1.27E-01	***-0.202***
Smg8	18	**0.055**	0.024	0.087	0.016	3.50	4.62E-04	4.43E-02	3.77	0.039	0.022	1.79	7.74E-02	1.14E-01	0.016
Rps20	18	**0.082**	0.036	0.128	0.023	3.50	4.71E-04	4.46E-02	5.96	**-0.097**	0.028	-3.47	7.97E-04	1.65E-03	-0.071
AI429214	16	**0.116**	0.051	0.182	0.033	3.48	4.96E-04	4.67E-02	1.79	0.073	0.078	0.93	3.53E-01	4.33E-01	-0.102
Rhou	18	**0.126**	0.055	0.198	0.036	3.48	5.00E-04	4.68E-02	5.21	-0.015	0.029	-0.51	6.10E-01	6.82E-01	-0.121
BC003965	18	**0.065**	0.028	0.103	0.019	3.46	5.48E-04	4.95E-02	4.67	0.014	0.021	0.66	5.10E-01	5.89E-01	-0.041

Table S4. Genes that were upregulated in the frontal cortex following sleep deprivation in our meta-analysis of public transcriptional profiling data (FDR<0.05, collective n=293) but not validated by a separate large RNA-Seq study (GSE114845, n=86 samples representing 222 mice). *Differential expression was considered to be not validated if 1) the opposite direction of effect was observed in GSE114845 than what was observed in the meta-analysis or 2) the same direction of effect was observed in GSE114845 as in the meta-analysis, but the differential expression was not significant (FDR<0.05). Column definitions are the same as* ***Table S3.***

Table S5: The full fast Gene Set Enrichment Analysis (fGSEA) results (10,436 gene sets). *This .xlsx file includes three worksheets: 1) The worksheet "fGSEA_Results_Directional" provides the full fGSEA results for the directional analysis, with each row representing the results for one gene set, and each column providing the fGSEA statistical output. The results are ordered by p-value, so that the top rows in the worksheet are the gene sets with the smallest p-values. 2) The*

worksheet "fGSEA_Results_Nondirectional" provides the full fGSEA results for the non-directional analysis in the same format as the directional analysis output. 3) The worksheet "ColumnDefinitions" provides the definitions for the variables present in each column in "fGSEA_Results_Directional" and "fGSEA_Results_NonDirectional".

Table S6. The full exploratory meta-analysis results (16,290 genes, 16,248 stable meta-analysis estimates). *This .xlsx file includes two worksheets: 1) The worksheet "ExploratoryMeta_FullResults" provides the full exploratory meta-analysis results, with each row representing the results for one gene, and each column providing either gene annotation or meta-analysis statistical output. The results are ordered by p-value for the effect of recovery sleep (RS), so that the top rows in the worksheet are the genes with the smallest p-values. 2) The worksheet "ColumnDefinitions" provides the definitions for the variables present in each column in "ExploratoryMeta_FullResults".*

	Current Meta-Analysis	GSE114845 Validation	Juszczak & Stankiewicz 2018	Jaszczyk et al., 2023			Jaszczyk et al., 2025	Meta-Analysis: Stankiewicz et al.., 2022			
	Sleep Deprivation		Responsive to Glucocorticoids	Acute 12h Corticosterone			Chronic Daily Corticosterone	Responsive to Stress			
**Gene**	**Log2FC**	**Validated?**	**Review of Literature**	**1 h post**	**5 h post**	**9 h post**	**Pooled: 5 days, 14 days, or 28 days**	**Overall Stress-responsive**	**Acute Stress-responsive**	**Medium Stress-responsive**	**Prolonged Stress-responsive**
Nr3c1	-0.122	Down	Down	Down	Down	Down	Down				
Car2	-0.112	No		Down	Down		Down				Down
Lrrc75a	-0.116	No	Down	Down	Down		Down				
Fa2h	-0.106	Down		Down	Down		Down	Up&Down			Up&Down
Septin8	-0.056	No		Down	Down	Down					
Herpud2	-0.051	Down		Down	Down	Down					
Cep120	-0.042	No		Down	Down	Down		Up&Down		Up&Down	
Zfp521	-0.108	Down		Down	Down		Down				
Sox21	-0.263	Down		Down	Down			Up&Down			Up&Down
Tbc1d31	-0.111	Down		Down	Down						
Smc2	-0.098	Down		Down	Down						
Il17rb	-0.093	Down		Down		Down					
Rufy2	-0.055	No		Down	Up&Down	Down					
Ahcy	-0.095	Down			Down	Down					
Rbm5	-0.073	Down			Down	Down					
Mpzl1	-0.112	Down		Down			Down				
Trim59	-0.169	Down		Down			Down				
2810459M11Rik	-0.157	Down		Down			Down				
Ablim3	-0.148	Down				Down					Up&Down
Vps26c	-0.081	Down						Up&Down	Down		
Mrtfa	0.102	Up		Up		Down	Up				
Card6	0.145	No		Up	Up	Down					
Ramp2	0.236	Up		Up	Up	Down					
Sorcs3	0.147	Up				Up				Up&Down	
Mchr1	0.095	Up		Up			Up				
Gata2	0.135	Up		Up			Up				
Vip	0.133	Up			Up		Up	Up&Down			Up&Down
Lingo3	0.155	Up		Up			Up				
Htr2a	0.158	Up		Up			Up				
Mink1	0.043	Up		Up	Up	Up				Down	
Smcr8	0.052	Up		Up						Up	
Nt5dc3	0.089	Up		Up	Up						
Hrob	0.103	No		Up		Up					
Hspa12b	0.153	Up		Up	Up	Up&Down					
Ppp1r10	0.102	Up		Up&Down	Up	Up					
Fam110b	0.112	Up	Up	Up&Down	Up	Up&Down					
Tsnax	0.064	Up			Up	Up					
Jund	0.083	No			Up	Up		Up&Down			
Fam78a	0.093	Up			Up	Up					
Rhou	0.126	No	Up	Up			Up				
Ociad2	0.147	Up		Up	Up		Up				
Arpc2	0.06	Up		Up	Up		Up				
Pip5k1a	0.043	Up		Up	Up		Up				
Kcng2	0.172	No		Up	Up		Up				
Zbtb16	0.255	Up		Up	Up	Down	Up	Up&Down			Up
Plcl2	0.057	Up		Up	Up	Up					
Wnk2	0.065	Up		Up	Up	Up					
Ddx51	0.084	Up		Up	Up	Up					
Gstm6	0.126	No		Up		Up		Up&Down		Up	
Prodh	0.123	Up		Up	Up	Up	Up				
Mapk4	0.095	Up		Up	Up	Up	Up				
Rhob	0.107	No	Up	Up	Up		Up				
Gm9925	0.189	Up		Up	Up	Up	Up				
Prr5	0.302	Up	Up	Up	Up		Up		Up&Down		
Ntrk2	0.047	Up	Up&Down	Up	Up	Up		Up&Down	Up		Up&Down
Myo1e	0.106	Up		Up	Up	Up				Up	
Mt2	0.349	Up	Up	Up	Up		Up	Up&Down	Up		

Table S7. Many of the genes that are differentially expressed in response to SD are also similarly responsive to glucocorticoids or stress. *We compared our list of DEGs for SD to a published database (Table S2 in (Juszczak et al., 2025)) overviewing evidence from two large studies examining the effects of acute (n=48) (Jaszczyk et al., 2023) and chronic daily (n=48)(Juszczak et al., 2025) stress hormone (corticosterone) exposure via drinking water on*

gene expression in the hippocampus, as well as previous evidence from a large vote-counting style meta-analysis of published effects of glucocorticoids (17 studies) (Juszczak and Stankiewicz, 2018) and of published effects of stress (79 animal studies (n=1887) and 3 human studies (n=121)) (Stankiewicz et al., 2022) on brain gene expression as measured using transcriptional profiling. We found that 78 of our DEGs have consistent evidence linking them to either glucocorticoid or stress effects on the brain. Of these, 57 DEGs showed effects of glucocorticoids or stress that were in the same direction as SD (shown above) and another 21 DEGs showed effects that were in the opposing direction from SD (not shown).

Supplemental Figures

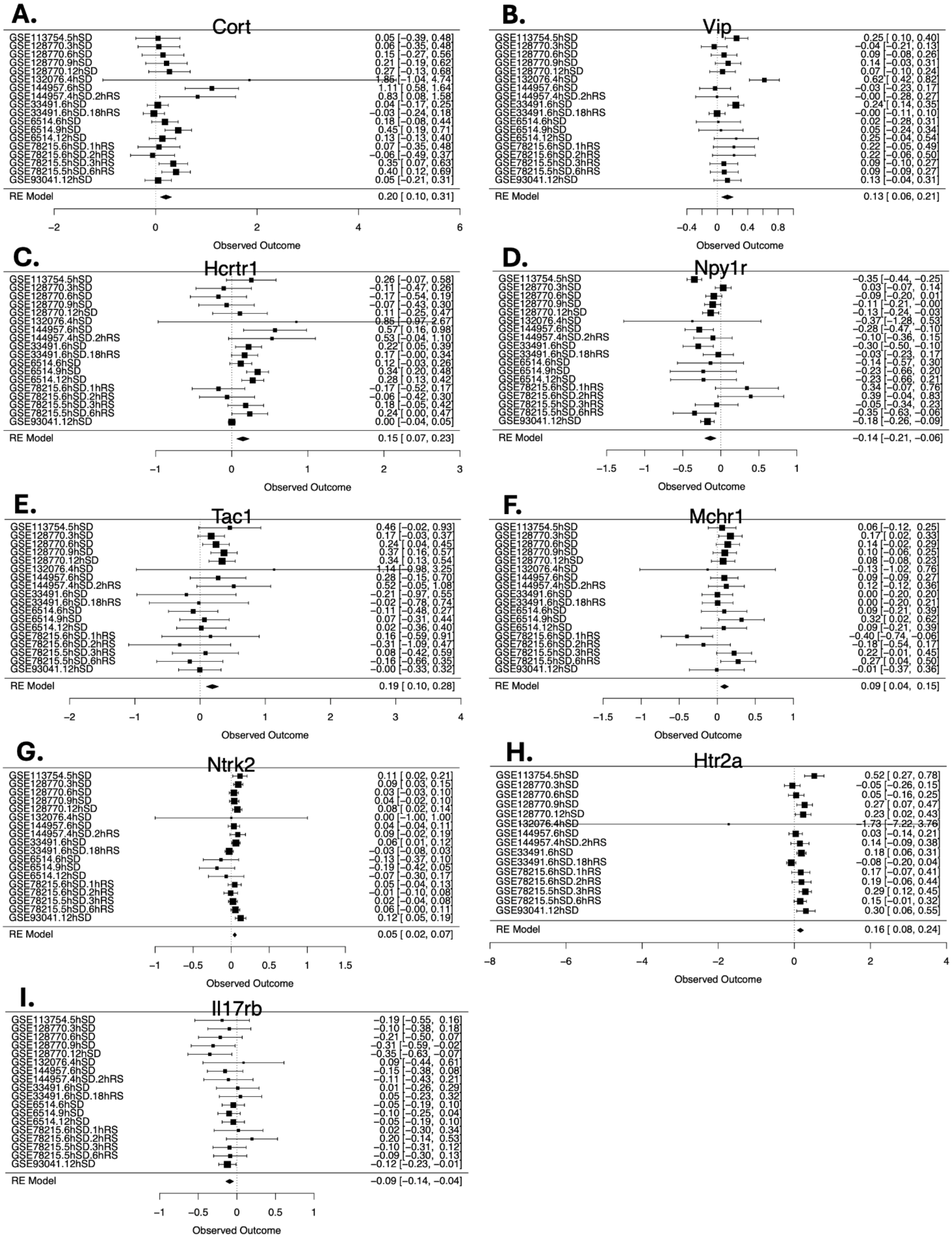

Figure S1. More example forest plots that show neuropeptide and cell signalling related genes that were consistently differentially expressed in the murine cortex across SD paradigms and

experiments in our meta-analysis of public datasets (collective n=293) and validated using an independent large RNA-Seq dataset (GSE114845). *Rows illustrate the effect sizes (SD vs. control Log2FC) with squares, with 95% confidence intervals (whiskers), for each of the datasets and the meta-analysis random effects meta-analysis model ("RE Model"). Each study is named using the duration of SD in hours (h), the Gene Expression Omnibus accession number (GSE#), and, if relevant, the duration of recovery sleep (RS) in hours (hr).*

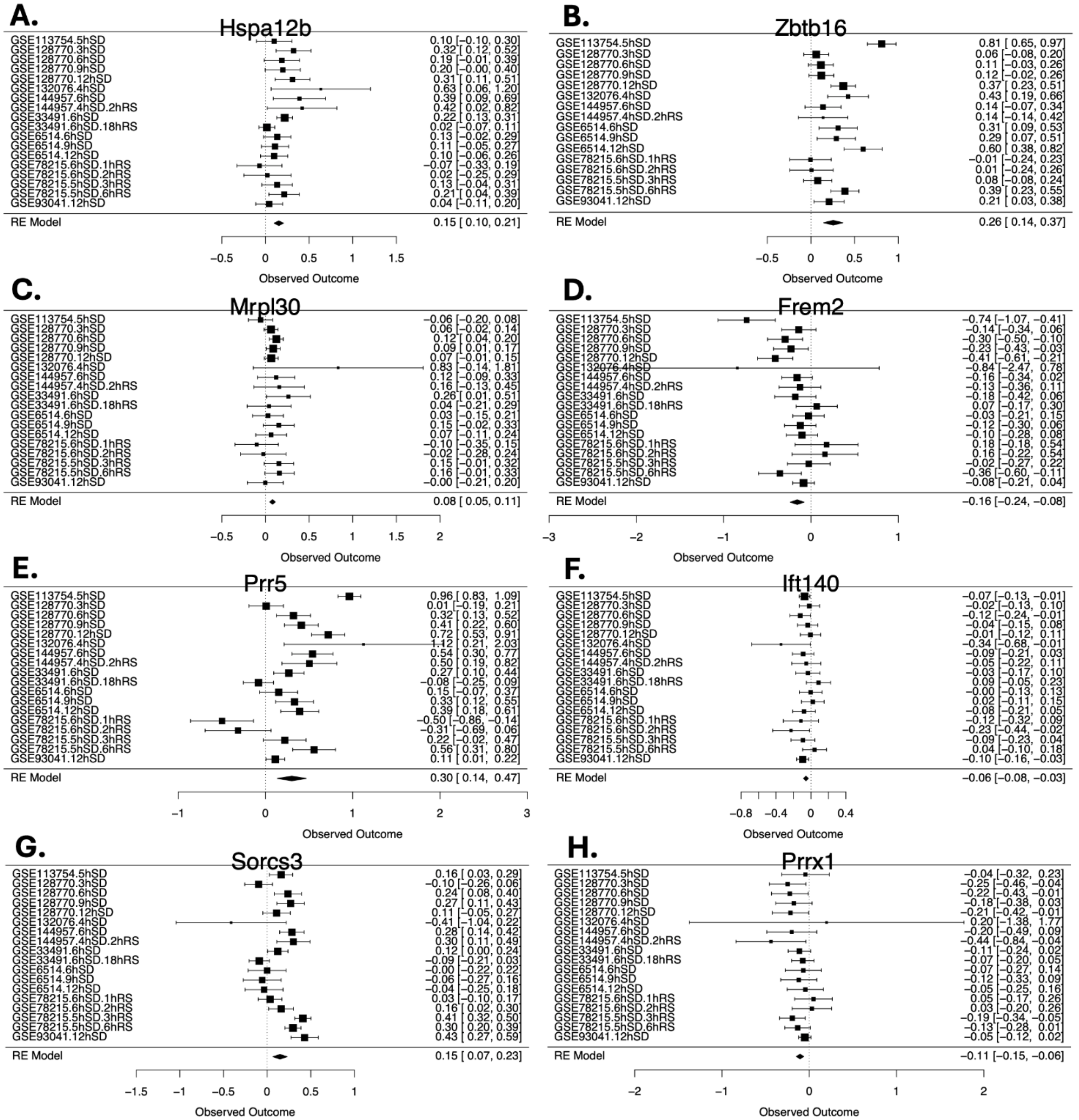

Figure S2. More example forest plots for other genes mentioned in the main text that were consistently differentially expressed in the murine cortex across SD paradigms and experiments in our meta-analysis of public datasets (collective n=293) and validated using an independent large RNA-Seq dataset (GSE114845). *Follows the conventions of* ***Figure S1****.*

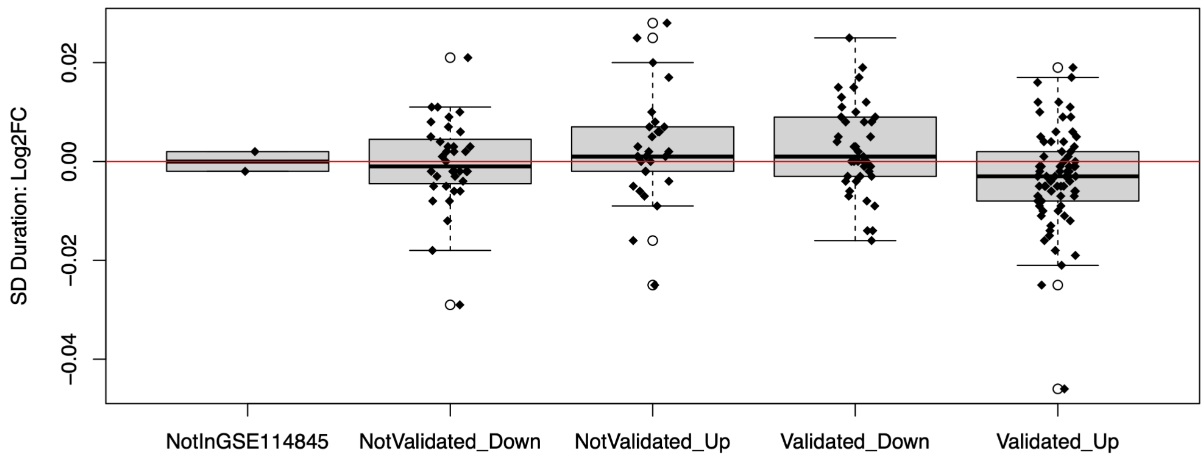

Figure S3. <u>Exploratory</u>: When examining the effects of sleep deprivation in the exploratory meta-analysis, the 182 genes that showed an effect of SD in the planned meta-analysis (FDR<0.05) did not consistently show an effect of SD Duration (Log2FC: y-axis) in the same direction as the effect of SD in the planned meta-analysis. *This was true for both the DEGs that were fully validated using GSE114845 and those that were not.*

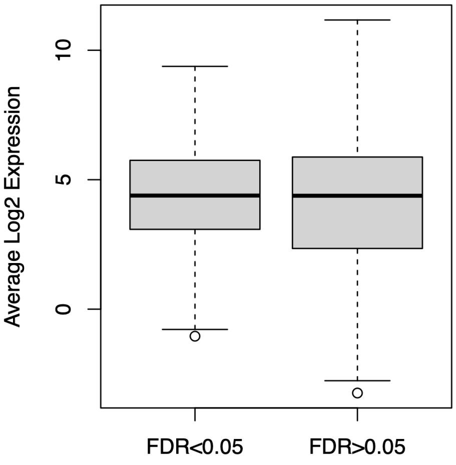

Figure S4. The genes that are consistently differentially expressed in response to sleep deprivation across datasets in the meta-analysis (FDR<0.05) do not tend to be biased towards genes with high level expression. *Referencing the average Log2 expression levels from the large RNA-Seq study that we used as validation for the meta-analysis (GSE114845), we did not find that the genes that were identified as DEGs in our meta-analysis (FDR<0.05) had noticeably higher levels of expression than genes that were not differentially expressed in our meta-analysis, although there may be a slight skew away from the lowest end of the distribution (as would be expected). Likewise, the DEGs that were fully validated using GSE114845 (115 genes)*

had an average Log2 expression of 4.31+/-0.20 SE, and the DEGs that did not validate using GSE114845 (65 genes) had an average expression of 3.97+/-0.25 SE, showing a slight skew toward higher level expressed genes validating that was not statistically significant.
